# The confused cancer patient: a case of 5-fluorouracil–induced encephalopathy

**DOI:** 10.3747/co.v15i5.252

**Published:** 2008-10

**Authors:** W.Y. Cheung, R.A. Fralick, S. Cheng

**Affiliations:** * Department of Medical Oncology and Hematology, University of Toronto, Toronto, ON; † Division of Medical Oncology, Odette Cancer Center, Toronto, ON

**Keywords:** Colorectal cancer, 5-fluorouracil, oxaliplatin, chemotherapy, complications

## Abstract

The fluorinated pyrimidine 5-fluorouracil (5-fu) is an anticancer agent used in most adjuvant and palliative treatment regimens for colorectal cancer. Neurotoxicities are considered extremely rare side effects of 5-fu. Here, we report a case of 5-fu–induced encephalopathy, manifesting as seizures and delirium, in an era of oxaliplatin-containing chemotherapy. If ammonia levels are elevated, lactulose may be considered in the initial management of neuropsychiatric complications from 5-fu.

## 1.CASE DESCRIPTION

A 53-year-old Korean man who had undergone curative surgical resection for stage iii (T3N2M0) colon cancer was evaluated in clinic by his medical oncologist before the start of adjuvant chemotherapy. He appeared clinically well on assessment and proceeded to receive his first cycle of modified folfox-6 chemotherapy. This regimen consisted of oxaliplatin 85 mg/m^2^, folinic acid 400 mg/m^2^, and 5-fluorouracil (5-fu) 400 mg/m^2^ bolus on day 1, followed by 5-fu 2400 mg/m^2^ by continuous infusion over the next 46 hours. Dexamethasone 12 mg and ondansetron 8 mg were given as pre-medication before chemotherapy.

The patient experienced no acute side effects or complications in the chemotherapy unit. He was discharged home with an ambulatory 5-fu pump and was scheduled to return to the clinic at completion of his 5-fu infusion.

Twenty-four hours later, the patient presented to the emergency department with new symptoms of erratic behaviour and confusion. Collateral history from the accompanying family members revealed that he had suffered a brief generalized tonic–clonic seizure earlier in the day, but no apparent history of head injuries, infectious symptoms, or recent alcohol intake was present. The patient’s other medical problems included well-controlled hypertension, hyperlipidemia, polycystic kidney disease, chronic renal insufficiency (baseline creatinine: 172 mmol/L), and stable chronic hepatitis B carrier status without laboratory or radiologic evidence of cirrhosis. No history of epilepsy was present. The patient’s only oral medications were amlodipine, quinapril, hydrochlorothiazide, and atorvastatin. He was an ex-smoker, and he denied alcohol abuse and illicit drugs.

On examination in the emergency department, the patient was neither alert nor oriented. He was unable to follow commands, and his verbal responses were incomprehensible. He was agitated, and his limbs were tremulous at rest. Temperature was 35.9°C; heart rate, 82/min; respirations, 18/min; blood pressure, 130/84 mmHg; and O_2_ saturation on room air, 99%. The patient’s eyes appeared deviated to his left side, but pupils were equal and reactive. Spontaneous twitching of the extremities, on the left side more than on the right, was noted and attributed to possible complex partial seizures. No other focal neurologic deficits or evidence of trauma were present. The Brudzinski and Kernig signs were negative. Examinations of chest, heart, and abdomen were otherwise unremarkable. The patient was noted to be incontinent of urine during the assessment. He was treated with intravenous diazepam and phenytoin (given in a loading dose of 1000 mg) for his agitation and presumed seizure activity respectively. At this point, his 5-fu infusion pump was still attached and functioning.

Laboratory investigations showed leucocytosis (white blood cell count: 19.5 × 10^9^/L) with left shift, but an otherwise normal complete blood cell count. Liver enzyme tests were within normal limits. Serum creatinine and blood urea nitrogen were elevated (239 mmol/L and 8.6 mmol/L respectively). Metabolic acidosis with an anion gap of 29 mmol/L was present. Imaging of the head and brain by computed tomography and magnetic resonance imaging scan respectively did not demonstrate evidence of infarction, hemorrhage, mass, or other abnormalities. Cerebrospinal fluid analysis was benign, and subsequent cultures of cerebrospinal fluid, blood, and urine were all negative. Of note, serum ammonia, at 316 mmol/L, and serum lactate, at 8.3 mmol/L, were elevated. The remainder of the toxicology screen was completely unremarkable. Vitamin B_12_ level and thyroid function tests were normal. An electroencephalogram showed a generalized slowing pattern consistent with metabolic encephalopathy.

Over the next 8 hours, the patient’s mental status progressively deteriorated to a comatose state. The 5-fu infusion pump was discontinued. Because the patient was no longer adequately protecting his airway, the critical care team was consulted for patient intubation and admission to the intensive care unit. With an apparent cause for the rapidly progressive cognitive dysfunction still lacking, lactulose at a dose of 30 mL every 4 hours was administered by nasogastric tube shortly thereafter in an effort to correct the patient’s encephalopathy. Phenytoin was continued.

Following the cessation of 5-fu and administration of lactulose, the patient gradually recovered over the next 48 hours from his acute confusional state. He experienced significant fatigue and muscle weakness, but no further seizures. Successive ammonia levels showed a decreasing trend. The patient was extubated, and phenytoin was discontinued. This man was discharged home on day 8 with instructions to follow up with his medical oncologist the following week.

After discharge, the patient required several weeks to recuperate before he could embark on additional chemotherapy. Considering his presumed neuropsychiatric complication from folfox chemotherapy, raltitrexed (3 mg/m^2^) and oxaliplatin (130 mg/m^2^) were given every 3 weeks as adjuvant treatment instead. This therapy was provided on an inpatient basis because of the insistence of the patient. No significant adverse reactions were noted. Unfortunately, re-staging investigations shortly thereafter revealed three metastatic lesions in the liver. We plan to continue with further chemotherapy for this patient with the goal of potential resection of his liver lesions in the future.

## 2.DISCUSSION AND CONCLUSIONS

Colorectal cancer (crc) is a leading cause of cancer morbidity and mortality in the developed world 1. In Canada alone, approximately 20,000 new cases and 8500 deaths occur each year 2. The primary treatment modality for localized crc is surgical resection, but more than 40% of stage ii and iii patients will still develop disease recurrence within 5 years of initial diagnosis 3,4. Therefore, adjuvant systemic therapy is offered to high-risk patients after surgery to eradicate occult micrometastatic disease and to reduce the risk of disease recurrence. Recent introductions of novel cytotoxic (for example, oxaliplatin) and molecularly targeted agents (for example, bevacizumab) into traditional 5-fu–based chemotherapy regimens have resulted in improved disease-free and overall survival in advanced disease, but at a cost of increased risk for toxicities 5,6.

The fluorinated pyrimidine 5-fu is an anti-metabolite and anti-neoplastic agent that has been used to treat a variety of cancers, including those of colon, rectum, breast, stomach, and pancreas. Although first introduced in 1958, 5-fu continues to be the cornerstone of current adjuvant chemotherapy protocols in crc namely the Mayo and the folfox regimens 7. Common side effects of 5-fu are bone marrow suppression leading to neutropenia and infections, and gastrointestinal toxicities such as stomatitis, nausea, vomiting, and diarrhea. Other less frequent adverse effects include alopecia, photosensitivity, and rarely, cardiotoxicity.

Albeit uncommon, 5-fu administration can also cause both acute and delayed neurotoxicities. The acute form typically presents as encephalopathy or a cerebellar-type syndrome. The delayed variety usually manifests as a subacute multifocal leucoencephalopathy. Seizures such as those demonstrated by the patient in the current case are an unusual finding and have only rarely been reported 8.

The precise mechanisms that underlie the various neurotoxicities are poorly understood, although several theories have been proposed. Some researchers believe that a non-specific immune-mediated process is responsible, but others suggest a more simplistic model in which ammonia, a product of 5-fu metabolism, accumulates in large amounts after either high-dose or continuous 5-fu administration 9,10.

The diagnosis of 5-fu–related encephalopathy is really one of exclusion. An astute clinical suspicion for this condition among cancer patients is therefore essential if early detection and prompt treatment are to occur. Although the finding of an increased ammonia level following chemotherapy administration supports the diagnosis, other metabolic and structural causes of altered mental status such as hypoglycemia, electrolyte disturbances, renal or hepatic failure, sepsis, medication intoxication or withdrawal, and brain metastases must first be excluded, as occurred in this case. Although our patient’s creatinine and blood urea nitrogen were both elevated, neither reached a level at which uremic encephalopathy would be anticipated. In addition, the patient’s leucocytosis was the probable result of his seizure activity, given that his infectious disease workup was entirely negative.

Discontinuation of 5-fu is a mainstay in the initial treatment of 5-fu encephalopathy. However, the optimal “antidote” and appropriate steps in the subsequent management of this condition remain unclear. Proposed treatment modalities have varied considerably in the literature, ranging from purely supportive measures to the use of corticosteroids or thiamine (or both), which, alone or in combination, have shown no consistent efficacy 8,11. In our case, the elevated ammonia level prompted a trial of lactulose administered in a manner similar to that used in hepatic encephalopathy. This maneuver, in combination with 5-fu cessation, resulted in timely symptomatic improvement in our patient.

The present case is also unique in uncovering the first patient to develop 5-fu encephalopathy as a result of folfox chemotherapy, which, over the past few years, has quickly become the new standard of care in the adjuvant and palliative therapies for crc. Because of current advances in screening and diagnostic strategies, a growing number of crc patients will be diagnosed at a stage of disease that necessitates chemotherapy. Although the rate of 5-fu neurotoxicity is low (<1%), the increasingly prevalent use of folfox chemotherapy may influence the absolute incidence of this complication 12. Further research is needed to better delineate and understand the mechanisms that contribute to 5-fu encephalopathy, but a high index of suspicion for this entity is warranted until more definitive diagnostic and therapeutic approaches are available. In the meantime, supportive care and empiric use of lactulose should be considered during management.

## References

[1] Jemal A, Siegel R, Ward E (2006). Cancer statistics, 2006. CA Cancer J Clin.

[2] (2006). Canadian Cancer Society and the National Cancer Institute of Canada. Canadian Cancer Statistics 2006.

[3] Minsky BD, Mies C, Rich TA, Recht A, Chaffey JT (1988). Potentially curative surgery of colon cancer: patterns of failure and survival. J Clin Oncol.

[4] Willett CG, Tepper JE, Cohen AM, Orlow E, Welch CE (1984). Failure patterns following curative resection of colonic carcinoma. Ann Surg.

[5] Andre T, Boni C, Mounedji–Boudiaf L (2004). Oxaliplatin, fluorouracil, and leucovorin as adjuvant treatment for colon cancer. N Engl J Med.

[6] Hurwitz H, Fehrenbacher L, Novotny W (2004). Bevacizumab plus irinotecan, fluorouracil, and leucovorin for metastatic colorectal cancer. N Engl J Med.

[7] Bosch L, Harbers E, Heidelberger C (1958). Studies on fluorinated pyrimidines. V. Effects on nucleic acid metabolism *in vitro*. Cancer Res.

[8] Pirzada NA, Ali I, Dafer RM (2000). Fluorouracil-induced neurotoxicity. Ann Pharmacother.

[9] Moore DH, Fowler WC, Crumpler LS (1990). 5-Fluorouracil neurotoxicity. Gynecol Oncol.

[10] Koenig H, Patel A (1970). Biochemical basis for fluorouracil neurotoxicity. The role of Krebs cycle inhibition by fluoroacetate. Arch Neurol.

[11] Cossaart N, SantaCruz KS, Preston D, Johnson P, Skikne BS (2003). Fatal chemotherapy-induced encephalopathy following high-dose therapy for metastatic breast cancer: a case report and review of the literature. Bone Marrow Transplant.

[12] Kim YA, Chung HC, Choi HJ, Rha SY, Seong JS, Jeung HC (2006). Intermediate dose 5-fluorouracil–induced encephalopathy. Jpn J Clin Oncol.

